# Heart Failure Duration Combined with Left Atrial Dimension Predicts Super-Response and Long-Term Prognosis in Patients with Cardiac Resynchronization Therapy Implantation

**DOI:** 10.1155/2019/2983752

**Published:** 2019-06-24

**Authors:** Zhinian Guo, Xiaoyan Liu, Chuan Liu, Jie Yang, Xiaofeng Cheng, Yunlong Chen, Ping Li, Yongming He, Jiang Wang

**Affiliations:** Department of Cardiology, Xinqiao Hospital, Army Medical University (Third Military Medical University), 183 Xinqiao Street, Chongqing 400037, China

## Abstract

**Background:**

Response to cardiac resynchronization therapy (CRT) varies significantly among patients. This study aimed to identify baseline characteristics that could predict super-response to CRT and to evaluate the long-term prognosis in super-responders.

**Methods:**

We retrospectively reviewed the data of 73 consecutive patients who received CRT. Patients were considered as super-responders after 6-month follow-up when NYHA class reduction to I or II combined with left ventricular ejection fraction (LVEF) ≥ 50% was observed. Patients were divided into super-responders group and non-super-responders group. All-cause mortality or hospitalization for heart failure (HF) was referred to the combined end point.

**Results:**

17 (23.3%) patients were super-responders. HF duration, left atrial dimension (LAD), and left bundle branch block (LBBB) were independent predictors of super-response to CRT. The combination of HF duration and LAD could provide more robust prediction of super-response than standalone HF duration (0.899 versus 0.789, Z = 2.207, P = 0.027) or standalone LAD (0.899 versus 0.775, Z = 2.487, P = 0.013). super-responders had excellent LV reverse remodeling. The cumulative incidences of combined end point were significantly lower in the super-responders group, LAD ≤ 42mm group, and combination of HF duration ≤ 48 months and LAD ≤ 42mm group. LBBB remained associated with a lowered risk of the combined end point (HR: 0.19, 95% CI: 0.07-0.57, P = 0.003), whereas LAD was associated with a raised risk of the combined end point (HR: 1.09, 95% CI: 1.02-1.17, P = 0.014).

**Conclusions:**

HF duration, LAD, and LBBB independently predicted super-response. The combination of HF duration and LAD makes more robust prediction of CRT super-response. Super-responders had excellent LV reverse remodeling and decreased the incidences of the combined end point. LBBB and LAD were independently associated with the combined end point.

## 1. Introduction

Cardiac resynchronization therapy (CRT) has become standard therapy for heart failure (HF) patients with left bundle branch block (LBBB) and prolonged QRS duration except optimum medical therapy [1]. It not only improves cardiac function, HF symptoms, and quality of life, but also reduces morbidity and mortality [2, 3]. However, response to CRT varies significantly among patients. Unfortunately, 30%-45% of patients fail to demonstrate improvement in cardiac function, left ventricular function (LVEF), or left ventricular (LV) remodeling after CRT[2]. Nevertheless, certain patients realize near normalization or normalization of LVEF, referred to as “super-responders”, was observed in approximately 12%-37.8% of CRT recipients [3–6].

Hence, it is of clinical importance to identify baseline characteristics which are predictive of super-response. It has been reported that shorter duration of HF symptoms, smaller LV, smaller left atrial dimension (LAD), LBBB, wider QRS, and nonischemic cardiomyopathy (NICM) are associated with super-response [5, 7, 8]. However, the definitions of super-response vary in previous studies, with different predictors of super-response to CRT reported.

The aim of the present retrospective study is (1) to identify baseline characteristics that could predict super-response to CRT, (2) to evaluate the value of independent predictors in predicting super-response, and (3) to analyze the long-term prognosis in super-responders.

## 2. Materials and Methods

### 2.1. Study Patients

This single center, retrospective study enrolled 73 consecutive patients who received CRT with or without an implantable defibrillator (CRT-P/D) from June 2014 to December 2017. The inclusion criteria were as follows: (1) New York Heart Association (NYHA) classes II to IV despite optimal medical therapy [angiotensin-converting-enzyme inhibitors (ACE-I) or angiotensin receptor blockers (ARB), *β*-blockers, and aldosterone antagonists] ≥ 3 months, (2) left ventricular ejection fraction (LVEF) ≤ 35%, and (3) QRS duration ≥ 130 ms. Patients with narrow QRS, myocardial infarction ≤ 3 months previously, or optimal medical therapy ≤ 3 months were excluded from this study. The study was approved by the Clinical Research Ethics Board at the Third Military Medical University. All participants volunteered to participate in the study and signed informed consent.

### 2.2. Echocardiography

Echocardiography was performed at baseline and after 6-month follow-up using the commercially available system (Vivid 7, General Electric-Vingmed, USA). Images were obtained using a 3.5 MHz transducer at an appropriate depth. The LVEF was calculated using the biplane Simpson's method from conventional apical four-chamber images. LV end-diastolic diameter (LVEDD) and left atrial diameter (LAD) were measured from M-mode. The area of mitral regurgitation (MR) was assessed semiquantitatively.

### 2.3. CRT Implantation

CRT was performed with standard techniques via the left subclavian vein. It was a routine procedure to insert the transvenous LV pacing lead into a branch of the coronary sinus. The LV lead was preferentially placed into the lateral or posterolateral vein or an alternative vein if the lateral or posterolateral vein could not be accessed. In summary, the position of the LV lead was determined by the coronary sinus angiographic data. Wherever the LV lead was placed, it was ensured that there were satisfactory pacing parameters without phrenic nerve stimulation. The right atrial lead was placed in the right atrial appendage and the right ventricular lead was placed in the right ventricular apex conventionally.

### 2.4. Patients' Follow-Up, Determination of Super-Response, and End Points

First follow-up visit was scheduled 1 month after CRT implantation and every 6 months thereafter. Echocardiography and HF symptoms were assessed at 6-month follow-up. Patients were considered as super-responders after 6-month follow-up when NYHA class reduction to I or II combined with near normalization of LVEF (defined as LVEF ≥ 50%) was observed. All-cause mortality or hospitalization for heart failure was referred to combined end point.

### 2.5. Statistical Analysis

Continuous variables were expressed as mean ± standard deviation or medians (25th–75th percentile) according to their normality following Kolmogorov–Smirnov test. Categorical variables were presented as count and percentage. Differences between groups were analyzed using Student's t-test for parametric variable, Mann-Whitney* U* test for nonparametric variables, and chi-square test for categorical variables. Intergroup comparisons were performed with paired t-test. Only variables with P < 0.1 in the univariate logistic regression were entered into multivariate logistic regression analysis to identify independent predictors of super-response using backward stepwise procedure. Receiver operator characteristic (ROC) curve was used for evaluation of continuous variables to predict super-response, and the Youden index was used to determine the optimal cut-off value. Kaplan-Meier curves, with Log Rank P test, compared all-cause death or HF hospitalization. Multivariate Cox proportional hazards models were constructed to identify independent predictors of combined end point, using the variables with P < 0.1 in the univariate Cox proportional hazards models. A two-sided P value < 0.05 was considered statistically significant. SPSS 19.0 (SPSS Inc., USA) and MedCalc 18.6.0 (MedCalc Inc., Belgium) were used to perform the statistical analysis.

## 3. Results

### 3.1. Baseline Characteristics of the Study Patients

In the present study, a total of 73 patients (21 females; mean age 60.32 ± 9.78 years) who fulfilled the inclusion criteria were enrolled. 46 patients had LBBB, 2 patients had right bundle branch block (RBBB), and 16 patients had intraventricular conduction delay (IVCD). Moreover, 1 patient upgraded from dual-chamber pacemaker (DDD) and 2 patients replaced CRT. Of all patients, 17 patients (23.3%) were considered as super-responders (NYHA class I or II, LVEF ≥ 50%); others were therefore regarded as non-super-responders. There were no significant differences with regard to age, gender, NYHA class, hypertension, diabetes, atrial fibrillation, ischemic etiology, LVEF, LVEDD, MR, QRS duration, ACE-I/ARB, and beta-blockers as well as spironolactone between the two groups. However, the super-responders group had shorter HF duration (P < 0.001) and smaller LAD (P = 0.001) and contained more frequent LBBB (P = 0.020) compared with the non-super-responders group (Table 1).

### 3.2. Predictors of Super-Response to CRT-P/D

As shown in Table 2, HF duration, LAD, and LBBB at baseline were associated with super-response to CRT-P/D in univariate analysis. Multivariate analysis including these variables and MR (univariate analysis, P < 0.1) revealed that HF duration (OR: 0.95, 95% CI: 0.92-0.98, P = 0.002), LAD (OR: 0.72, 95% CI: 0.58-0.89, P = 0.002), and LBBB (OR: 10.91, 95% CI: 1.30-91.64, P = 0.028) were independent predictors of super-response to CRT-P/D.

**Figure 1:**
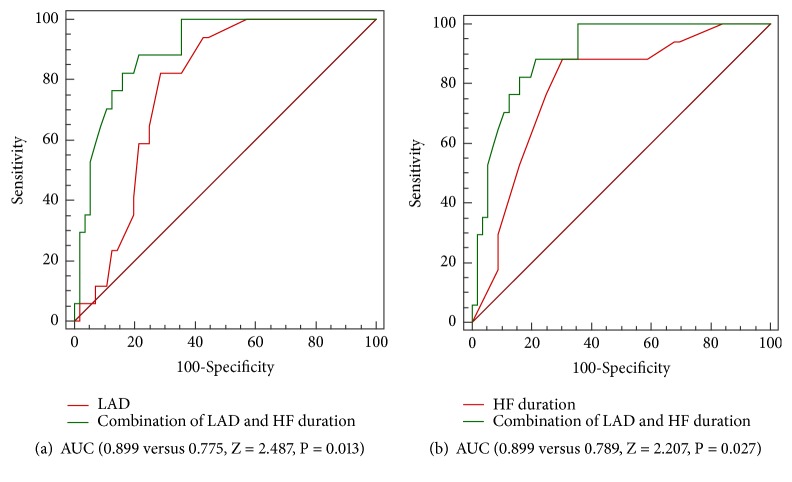
(a) ROC curve for LAD (red line) and combination of HF duration and LAD (green line) in predicting super-response; (b) ROC curve for HF duration (red line) and combination of HF duration and LAD (green line) in predicting super-response. ROC: receiver operator characteristic; HF: heart failure; LAD: left atrial dimension; AUC: the area under the curve.

Receiver operating characteristic (ROC) curve analysis was performed to evaluate the value of HF duration, LAD and LBBB in predicting super-response to CRT. The area under the curve (AUC) for baseline HF duration was 0.789 (95% CI: 0.67-0.91, P < 0.001), with HF duration ≤ 36 months having 88% sensitivity and 70% specificity. Baseline LAD ≤ 42 mm was 82% sensitive and 71% specific for predicting super-response to CRT (AUC 0.775; 95% CI: 0.67-0.88; P < 0.001). For combination of HF duration and LAD, the AUC was 0.899 (95% CI: 0.83-0.98, P < 0.001), with HF duration ≤ 48 months together with LAD ≤ 42 mm having 88% sensitivity and 79% specificity. There was no significant difference between the AUC of HF duration and LAD (0.789 versus 0.775, Z = 0.190, P = 0.849). Combination of HF duration and LAD significantly increased the AUC compared with standalone HF duration (0.899 versus 0.789, Z = 2.207, P = 0.027) or standalone LAD (0.899 versus 0.775, Z = 2.487, P = 0.013) (Figure 1, Table 3).

### 3.3. Six-Month Follow-Up after CRT

Three patients died within 6 months after CRT in the non-super-responders group. Therefore, a total of 70 patients completed the echocardiographic, clinic visit, or telephone surveys at 6-month follow-up. Patients' characteristics including cardiac function and echocardiographic data at baseline and follow-up in two groups are summarized in Table 4. After 6-month follow-up, there were significant improvements of NYHA class, LVEF, LVEDD, and MR in both groups (P < 0.05 for all comparisons). Compared with non-super-responders group, the super-responders group demonstrated significantly better outcome as indicated by better improvement in cardiac function (P < 0.001), higher LVEF (P < 0.001), smaller LVEDD (P < 0.001), and MR (P < 0.001) at 6-month follow-up.

### 3.4. Long-Term Prognosis

**Figure 2:**
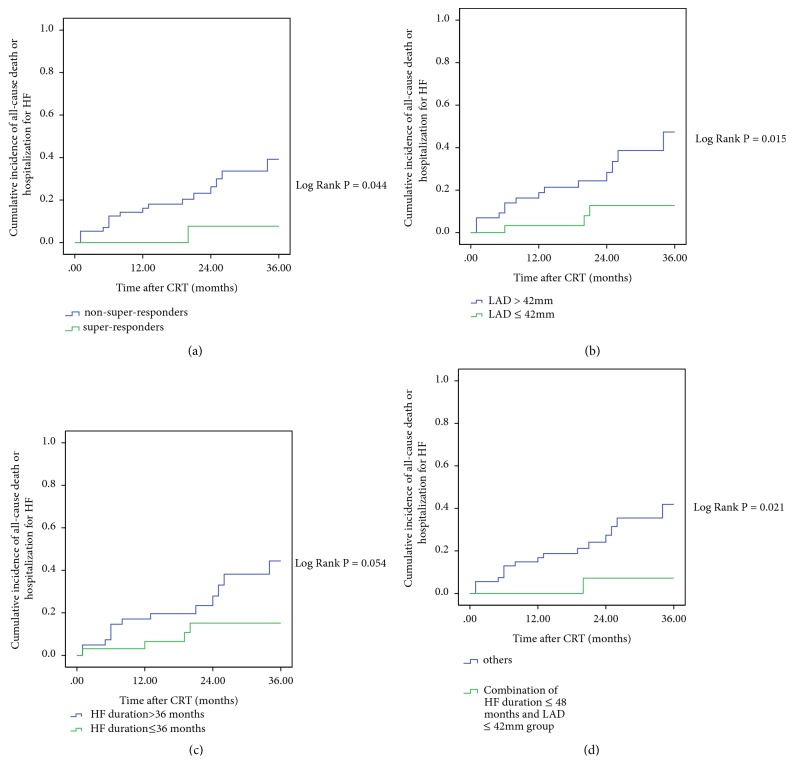
Four Kaplan-Meier curves (response category, LAD, HF duration, and combination of HF duration and LAD) of cumulative incidence of all-cause death or hospitalization for heart failure. (a) For the combined end point, super-responders (green line) performed the best compared to non-super-responders (blue line). (b) For the combined end point, LAD ≤ 42mm group (green line) performed the best compared to LAD > 42mm group (blue line). (c) For the combined end point, there was no difference between HF duration ≤ 36 months group and HF duration > 36 months group. (d) For the combined end point, combination of HF duration ≤ 48 months and LAD ≤ 42mm group (green line) performed the best compared to others (blue line). HF: heart failure; LAD: left atrial dimension.

During a mean follow-up of 22.56 ± 10.38 months, the combined end point of hospitalization for HF or all-cause death occurred in 17 patients. In the super-responders group, 1 (5.9%) hospitalization for HF and 0 (0%) deaths were reported, compared with 10 (17.9%) hospitalizations for HF and 6 (10.7%) deaths in the non-super-responders group. As shown in the Kaplan-Meier curves (Figure 2), the cumulative incidences of combined end point were significantly lower in the super-responders group, LAD ≤ 42mm group, and combination of HF duration ≤ 48 months and LAD ≤ 42mm group compared with the non-super-responders group (Log Rank P = 0.044), LAD > 42mm group (Log Rank P = 0.015), and others (Log Rank P = 0.021), respectively. However, no statistically significant difference was observed between the HF duration ≤ 36 months group and the HF duration > 36 months group for the combined end point (Log Rank P = 0.054).

### 3.5. Predictors of the Combined End Point

A multivariate Cox regression model was carried out adjusting for LAD, LVEDD, and LBBB (univariate regression model analysis, P < 0.1). LBBB remained associated with a lowered risk of the combined end point (HR: 0.19, 95% CI: 0.07-0.57, P = 0.003), whereas LAD was associated with a raised risk of the combined end point (HR: 1.09, 95% CI: 1.02-1.17, P = 0.014) (Table 5).

## 4. Discussion

The main findings of our study were as follows: (1) HF duration, LAD, and LBBB were independent predictors of super-response to CRT; (2) the combination of HF duration and LAD could provide better prediction of CRT super-response; (3) super-responders had excellent LV reverse remodeling and clinical prognosis during follow-up; (4) the cumulative incidences of combined end point were significantly lower in the super-responders group, LAD ≤ 42mm group, and combination of HF duration ≤ 48 months and LAD ≤ 42mm group compared with the non-super-responders group, LAD > 42mm group, and others, respectively; (5) LBBB showed a decreased risk for combined end point, whereas LAD was associated with an increased risk of combined end point.

LVEF was used to diagnose HF and evaluate the prognosis of HF treatments. Reduced LVEF was associated with poor survival [9]. Moreover, most clinical trials and guidelines for CRT-P/D implantation are based on measurement of LVEF to select patients. Hence, LVEF was regarded as one of the most widely used measurement to define CRT super-response. In our study, the definition of super-response included the combination of NYHA class (I-II) and LVEF ≥ 50%. Previous studies reported an incidence of 12% to 37.8% of CRT super-response [9]. We found an incidence of 23.3% of CRT super-response based on our definition of super-response, similar to the previous studies by Castellant et al. (21.5%)[10] and Gasparini et al. (26%)[11] using the same definition.

In the MADIT-CRT study by Hsu et al. of 752 CRT recipients, smaller LA volume was independently associated with super-response to CRT therapy [5]. Similarly, Reant et al. demonstrated that LA volume <55 ml could independently predict CRT super-response. In our study, LAD could be independently predictive of super-response, aligned with the above two studies. Moreover, using ROC curves, LAD ≤ 42 mm had 82% sensitivity and 71% specificity for predicting super-response to CRT (AUC 0.775; 95% CI: 0.67-0.88; P < 0.001). We also found LAD ≤ 42mm was associated with improved clinical outcome and increased LAD tended to be an increased risk factor of the combined end point (HR: 1.09, 95% CI: 1.02-1.17, P = 0.014). LAD was a sensitive marker of chronic HF. Previous studies had reported that increased LA size was associated with cardiovascular morbidity and mortality in HF patients [12, 13]. In addition, LAD was correlated with changes in electrophysiological characteristics, such as interatrial conduction times and functional LA conduction blocks [8, 14]. Hence, enlarged LA limited positive effects of CRT and contributed progression of HF. In other words, better clinical outcome and response were associated with smaller LA. Further investigations were justified to clarify the pathophysiological mechanisms of enlarged LA influencing outcomes in patients receiving CRT and whether addressing these mechanisms could improve the response to CRT.

In the present study, HF duration was an independent predictor of super-response to CRT, with HF duration ≤ 36 months having 88% sensitivity and 70% specificity (AUC, 0.789, 95% CI: 0.67-0.91, P < 0.001), in line with a previous study which showed that HF duration ≤ 12 months remained significantly associated with super-response in multivariate analysis [15]. However, our present results showed that although there was no statistical difference, HF duration ≤ 36 months tended to decrease the occurrence of combined end point. To our knowledge, it was the first study to evaluate the value of combination of HF duration and LAD in predicting super-response. The result showed that the combination of HF duration and LAD could provide better performance than HF duration alone (0.899 versus 0.789, Z = 2.207, P = 0.027) or LAD alone (0.899 versus 0.775, Z = 2.487, P = 0.013) in prediction of super-response. It may indicate that CRT super-response could occur more easily in the earlier phases of HF. More researches were needed to confirm our results in the future.

Abnormal left ventricular activation sequence in LBBB might lead to electrical and mechanical dyssynchrony [16]. Thus, CRT targeting resynchronization of the delayed left ventricle was recommended for HF patients with LBBB [17]. The previous study, which enrolled 233 patients, 101 patients (43.3%) with LBBB, 68.8% (22) in super-responders, and 39.3% (79) in non-super-responders, reported that only LBBB was significantly associated with super-response in multivariate analysis [18]. Hsu et al. also found that LBBB could independently predict super-response to CRT therapy, which enrolled 752 patients, 534 patients (71.0%) with LBBB, 55.8% (106) in hyporesponders, 70.9% (263) in responders, and 86.4% (165) in super-responders[5]. In our study, 46 patients (63.0%) had LBBB, 88.2% (15) in super-responders and 55.4% (31) in non-super-responders. Similarly, we found that LBBB were independent predictors of super-response to CRT. In addition, a previous study by Tian et al. of 58 CRT recipients showed that true LBBB was an independent predictor of super-response [19]. Moreover, our study showed that LBBB was associated with a lowered risk of the combined end point (HR: 0.19, 95% CI: 0.07-0.57, P = 0.003), in line with the previous MADIT-CRT study by Hsu et al. which demonstrated that baseline LBBB was significantly associated with a decreased risk of HF or all-cause death (HR: 0.57; 95% CI: 0.34 to 0.94; p = 0.029) [5].

Several other variables, including female sex, smaller LV, NICM, BMI, and wider QRS duration, were reported to be associated with super-response to CRT. Unfortunately, those variables could not predict super-response in our study, which could be due to differences with the super-response definition adopted and patients enrolled.

In the study, super-responders had greater reductions in LVEDD (P < 0.001) and MR (P < 0.001) than non-super-responders. In other words, super-responders had excellent LV reverse remodeling, which was associated with favourable clinical outcome, in line with a previous study[20]. The incidences of all-cause death and HF hospitalization were 8.2% and 15.1%, respectively, in our study. In the MADIT-CRT study, all-cause death occurred in 3.3% of all patients and the incidence of nonfatal HF event was 6.4% with a median follow-up of 15.2 months [5]. In addition, one recent study with 347 patients showed all-cause death and HF hospitalization occurred in 23% and 22% in all patients who were followed up for a median of 5.3 years, respectively [4]. We found super-response was associated with a reduced occurrence of all-cause death or HF hospitalization, which was consistent with the previous studies [4, 5, 18, 20, 21]. Moreover, a previous study reported that the patients considered as super-responders had a significantly reduced incidence of ventricular arrhythmias [22]. Similarly, Killu et al. demonstrated that the super-response to CRT-D group had very low rates of ventricular arrhythmias requiring implantable cardioverter defibrillator (ICD) therapy compared with the non-super-response to CRT-D group [23].

## 5. Limitations

This study has a few limitations. First, the retrospective nature of this study probably resulted in unidentified confounders. Second, patients in our study came from a single center and the sample size was relatively small; therefore, it may not be sufficiently representative. Third, the duration of follow-up in our study was relatively short and therefore predictors of delayed improvement of LVEF could not be evaluated. Last, patients who were lost to follow-up were not included in our study, so there may be patient selection bias. However, those aspects were inherent limitations of the “real world” studies, despite our best efforts to avoid these limitations. Therefore, further large multiple-center prospective trials were needed to confirm our results in the future.

## 6. Conclusions

HF duration, LAD, and LBBB were independent predictors of super-response to CRT. The combination of HF duration and LAD strongly predicted CRT super-response. Super-responders had excellent LV reverse remodeling and clinical prognosis during follow-up. LAD ≤ 42mm and the combination of HF duration ≤ 48 months and LAD ≤ 42mm decreased the incidences of combined end point. LBBB and LAD were independently associated with all-cause death and HF hospitalization.

## Figures and Tables

**Table 1:** Baseline characteristics of patients between super-responders and non-super-responders.

characteristics	Total population	super-responders	non-super-responders	*P* Value
	(n=73)	(n=17)	(n=56)	
Age (years)	60.32 ± 9.78	60.00 ± 9.87	61.35 ± 9.72	0.621
Gender (female)	21 (28.8%)	7 (41.2%)	14 (25.0%)	0.197
NYHA class	3 (3-3)	3 (2-3)	3 (3-3)	0.285
HF duration (months)	48 (18-78)	12 (6-60)	60 (27-96)	*< 0.001*
Hypertension, n (%)	16 (21.9%)	5 (29.4%)	11 (19.6%)	0.394
Diabetes, n (%)	8 (11.0%)	3 (17.6%)	5 (8.9%)	0.378
Atrial fibrillation, n (%)	9 (12.3%)	1 (5.9%)	8 (14.3%)	0.675
Ischemic etiology, n (%)	12 (16.4%)	4 (23.5%)	8 (14.3%)	0.456
LVEF (%)	32 (27-34)	32.6 (28-35)	31 (25-33.75)	0.140
LAD (mm)	45 (39.75-47)	40 (38-42)	46 (41.23-48)	*0.001*
LVEDD (mm)	70.22 ± 7.98	68.25 ± 8.37	70.82 ± 7.84	0.247
MR (cm^2^)	7.0 (4.5-9.98)	5.99 (4.30-7.55)	7.32 (4.5-10.41)	0.079
QRS duration (ms)	164.0 ± 20.99	171.2 ± 16.54	161.9 ± 21.83	0.109
LBBB, n (%)	46 (63.0%)	15 (88.2%)	31 (55.4%)	*0.020*
ACE-I/ARB, n (%)	67 (91.8%)	16 (94.1%)	51 (91.1%)	1.000
Beta-blockers, n (%)	66 (90.4%)	16 (94.1%)	50 (89.3%)	1.000
Spironolactone, n (%)	61 (83.6%)	15 (88.2%)	46 (82.1%)	0.720

Values are mean ± SD, median (range), or n (%).

NYHA: New York Heart Association; HF: Heart Failure; LVEF: left ventricular ejection fraction; LAD: left atrial dimension; LVEDD: left ventricular end-diastolic dimension; MR: mitral regurgitation; LBBB: left bundle branch block; ACEI: angiotensin-converting-enzyme inhibitor; ARB: angiotensin receptor blocker.

**Table 2:** Univariate and multivariate logistic regression analyses of predictors of super-response.

	Univariate analysis		Multivariate analysis	
Baseline characteristics	OR (95% CI)	*P* Value	OR (95% CI)	*P* Value
Age	1.02 (0.96-1.07)	0.616		
Gender	2.10 (0.67-6.56)	0.202		
NYHA class	0.61 (0.25-1.49)	0.274		
HF duration	0.97 (0.94-0.99)	*0.003*	0.95 (0.92-0.98)	*0.002*
Hypertension	1.71 (0.50-5.86)	0.397		
Diabetes	2.19 (0.47-10.29)	0.322		
Atrial fibrillation	0.38 (0.04-3.23)	0.372		
Ischemic etiology	1.85 (0.48-7.10)	0.373		
LVEF	1.10 (0.96-1.26)	0.186		
LAD	0.83 (0.74-0.94)	*0.003*	0.72 (0.58-0.89)	*0.002*
LVEDD	0.96 (0.89-1.03)	0.246		
MR	0.88 (0.75-1.02)	0.081	1.21 (0.89-1.65)	0.223
QRS duration	1.02 (1.00-1.05)	0.115		
LBBB	6.05 (1.26-28.98)	*0.024*	10.91 (1.30-91.64)	*0.028*
ACE-I/ARB	1.57 (0.17-14.43)	0.691		
Beta-blockers	1.92 (0.22-17.17)	0.559		
Spironolactone	1.63 (0.32-8.29)	0.556		

NYHA: New York Heart Association; HF: heart failure; LVEF: left ventricular ejection fraction;

LAD: left atrial dimension; LVEDD: left ventricular end-diastolic dimension; MR: mitral regurgitation; LBBB: left bundle branch block; ACE-I: angiotensin-converting-enzyme inhibitor; ARB: angiotensin receptor blocker

OR: odds ratio; 95% CI: 95% confidence interval.

**Table 3:** Area under the curve and sensitivity and specificity for HF duration, LAD, and combination of HF duration and LAD.

Variables	AUC	95% CI	*P* for AUC	Cut-off value	Sensitivity (%)	Specificity (%)
HF duration (months)	0.789	0.67-0.91	*< 0.001*	≤ 36	88	70
LAD (mm)	0.775	0.67-0.88	*< 0.001*	≤ 42	82	71
Combination of HF duration and LAD (months, mm)	0.899	0.83-0.98	*< 0.001*	≤ 48 and ≤ 42	88	79

HF: heart failure; LAD: left atrial dimension; AUC: the area under the curve; 95% CI: 95% confidence interval.

**Table 4:** Comparison of cardiac function and echocardiographic data at baseline and follow-up in two groups.

Variables	super-responders (n=17)			non-super-responders (n=56)			*P∗* Value
	baseline	follow-up	*P* Value	baseline	follow-up	*P* Value	
NYHA class	3 (2-3)	2 (1-2)	*< 0.001*	3 (3-3)	2 (2-3)	*< 0.001*	*< 0.001*
LVEF (%)	32.6 (28-35)	57 (54-59)	*< 0.001*	31(25-33.8)	34.5(29.3-38.8)	*0.003*	*< 0.001*
LVEDD (mm)	68.3 ± 8.4	55.3 ± 6.1	*< 0.001*	70.8 ± 7.8	68.7 ± 9.5	*0.034*	*< 0.001*
MR (cm^2^)	6.0 (4.3-7.6)	1 (0-2.4)	*< 0.001*	7.3 (4.5-10.4)	4.6 (2.5-7.3)	*< 0.001*	*< 0.001*

NYHA: New York Heart Association; LVEF: left ventricular ejection fraction; LVEDD: left ventricular end-diastolic dimension; MR: mitral regurgitation.

*P∗*: follow-up between two groups.

**Table 5:** Multivariate Cox regression models for combined end point.

Variables	HR	95% CI	*P* Value
LAD (mm)	1.09	1.02-1.17	0.014
LBBB	0.19	0.07-0.57	0.003

LAD: left atrial dimension; LBBB: left bundle branch block.

95% CI: 95% confidence interval; HR: hazard ratio.

## Data Availability

The data used to support the findings of this study are available from the corresponding author upon request.
